# Distal Humerus as Delayed Site of Metastasis from Small Cell Carcinoma of Gallbladder

**DOI:** 10.1155/2013/946835

**Published:** 2013-12-14

**Authors:** Mutahir A. Tunio, Mushabbab AlAsiri, Asma Mohammed F. Ali, Eyad Fawzi AlSaeed, Muhammad Shuja, Hanadi Fatani

**Affiliations:** ^1^Radiation Oncology, Comprehensive Cancer Center, King Fahad Medical City, Riyadh 59046, Saudi Arabia; ^2^Medical Oncology, Comprehensive Cancer Center, King Fahad Medical City, Riyadh 59046, Saudi Arabia; ^3^Radiation Oncology, King Saud University, Riyadh 59046, Saudi Arabia; ^4^Department of Cytogenetics, Pathology, King Fahad Medical City, Riyadh 11525, Saudi Arabia

## Abstract

*Background*. Small cell carcinoma (SCC) of the gallbladder is a rare entity and is often seen in elderly women. SCC of gallbladder is typically a nonsecretory carcinoid tumor without overt clinical symptoms and is often discovered at advanced stages. SCC of gallbladder carries a dismal prognosis as compared to SCC of lung and adenocarcinoma of gallbladder. To date, only 73 case reports have been published in the world literature. *Case Presentation*. Herein, we report a case of a 73-year-old Saudi woman who presented with one week history of right upper quadrant abdominal pain and obstructive jaundice and was found to be a case of locally advanced, metastatic SCC of gallbladder cT4N1M1 (liver, para-aortic lymph nodes, and bone). The patient was treated with neoadjuvant etoposide and cisplatin (EP) chemotherapy three cycles after biliary stenting followed by radical cholecystectomy, lymphadenectomy, and adjuvant EP chemotherapy and then one year later developed distal humerus osseous metastasis. *Conclusion*. SCC of the gallbladder is very rare entity and is often seen at advanced stages. Osseous metastases of peripheral skeleton from SCC gallbladder are rarely reported. Surgery is curative option but only for early stage tumors. Incorporation of chemotherapy along with radical resection increases the survival.

## 1. Introduction

Gallbladder carcinomas are the fifth most common gastrointestinal malignancies [1]. Most common histologic type is the adenocarcinoma (99%) which occurs mainly in elderly women with possible risk factors (cholelithiasis, choledochal cysts or chronic cholecystitis) [2]. The gallbladder small cell carcinoma (SCC) is an extremely rare histologic type comprising 0.2% of all gastrointestinal carcinoids [3]. Contrary to other sites carcinoids, the gallbladder SCCs are generally nonfunctional tumors without paraneoplastic symptoms secondary to secretion of biologically active peptides [4]. First SCC of gallbladder was reported in 1981 by Albores-Saavedra et al. and after that 73 cases have been published in the literature till date [5]. Further, similar to gallbladder adenocarcinoma, SCC has a strong association with cholelithiasis [6]. Gallbladder mucosa is devoid of neuroectodermal cells and SCC arises from metaplastic epithelium of the gallbladder wall secondary to cholelithiasis and chronic cholecystitis [7]. SCC is often diagnosed at advanced stages (locally advanced or metastatic) and carries a grave prognosis with survival rates worse than gallbladder adenocarcinomas [8].

Herein, we report a case of a 73-year-old Saudi woman with locally advanced, metastatic SCC of gladder cT4N1 M1 (liver, para-aortic lymph nodes, and bone) who was treated with multimodality regimen (neoadjuvant and adjuvant chemotherapy followed by surgery).

## 2. Case History

**Figure 1:**
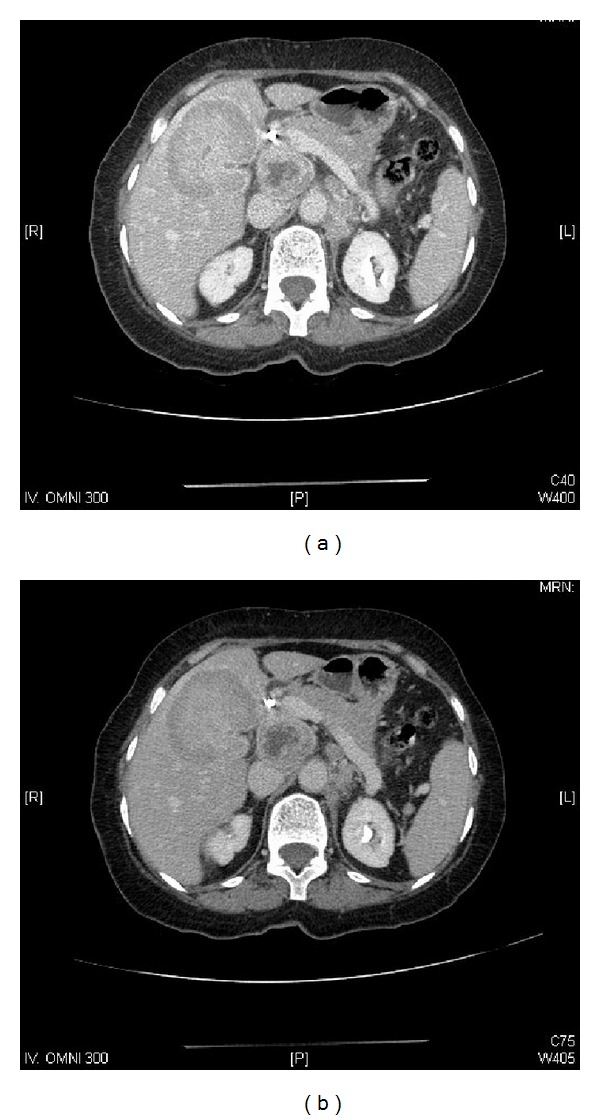
Prechemotherapy abdominal computed tomography (CT) showing (a) a large 7.9 × 5.1 cm heterogeneous mass involving the gallbladder and the adjacent liver and (b) enlarged portocaval and para-aortic lymph nodes.

**Figure 2:**
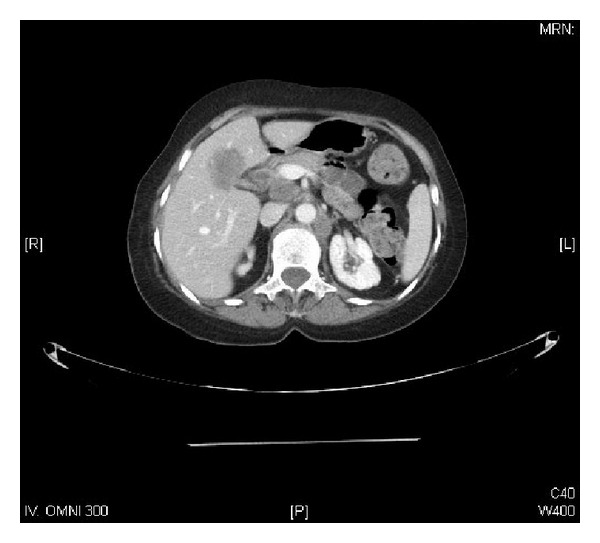
Postchemotherapy abdominal CT showing interval regression of the known masses around the GB, portocaval, aortocaval, and left paraaortic stations by about 35–45%.

**Figure 3:**
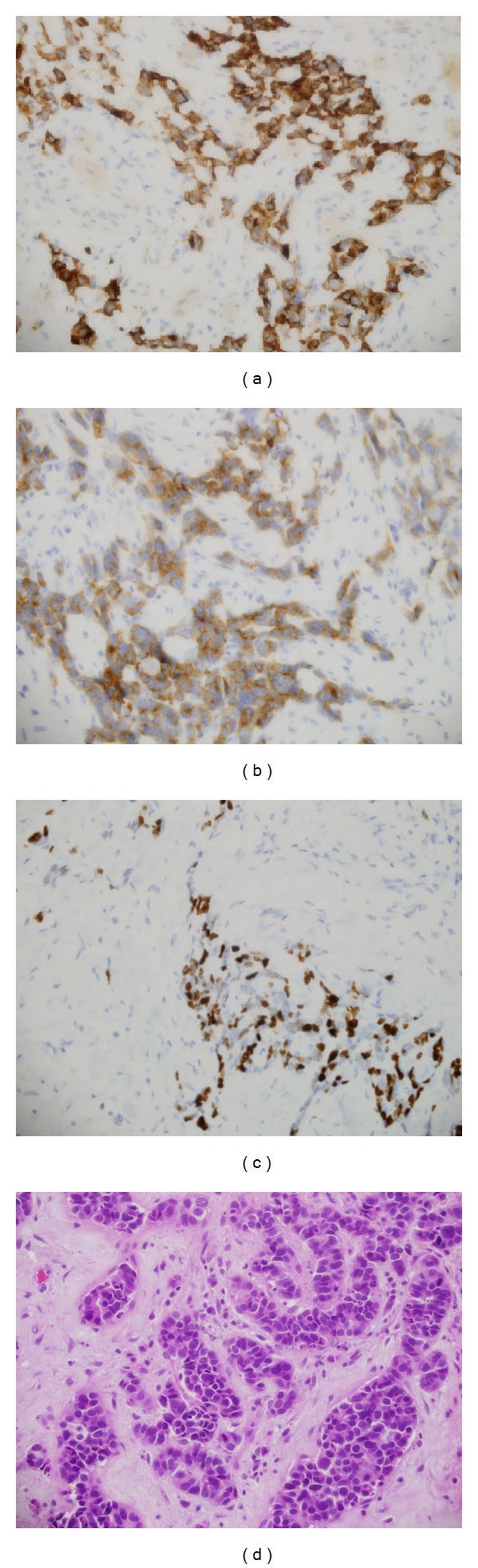
Histopathology of surgical specimen showing (a) chromogranin positivity, (b) synaptophysin positivity, (c) Ki-67 positivity, and (d) hematoxylin and eosin stained small cells consistent with small cell carcinoma of gallbladder.

**Figure 4:**
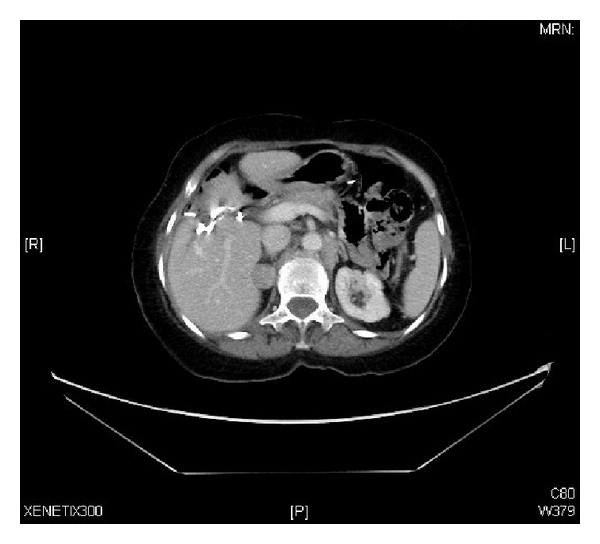
Follow-up abdominal CT at one year showing the recurrent soft tissue mass adjacent to the liver inseparable from the right adrenal and interval increased in size of the left para-aortic lymph nodes.

**Figure 5:**
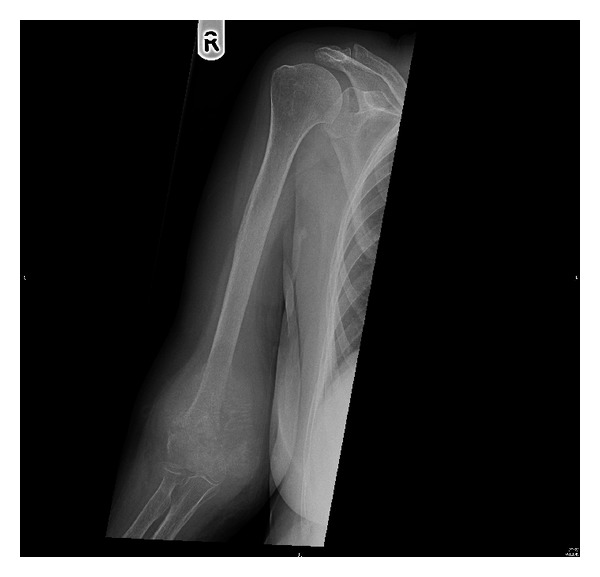
Plain radiograph of right arm showing aggressive bony lesion involving the distal metadiaphysis and epiphysis of the right humerus and associated with the sunburst periosteal reaction and pathological fracture.

A 73-year-old female presented to our clinic with one week history of epigastric and right upper quadrant abdominal pain, loss of appetite, and jaundice; however she denied any weight loss, nausea, or vomiting. She took some nonsteroidal anti-inflammatory analgesics (NSAIDs) but was not relieved. On physical examination, she was found to have good performance scale (ECOG-0) and severe icteric without any pallor or palpable lymphadenopathy. On abdominal examination, there was deep tenderness in right hypochondrium. The rest of systemic examination was found unremarkable. Baseline liver function tests were markedly deranged (total bilirubin 64.1 micromole/liter ↑ (*μ*mol/L), alkaline phosphatase 197 U/L ↑, and ALT 91 ↑); however, hematology and electrolytes were within normal limits. Ultrasonography revealed a gallbladder mass of size 1.5 cm in diameter along with dilatation of the intrahepatic biliary ducts with two hepatic masses of size 4.20 × 3.6 cm located in segment I of the liver and another hepatic mass measuring about 6.5 × 5 cm in segment IV of the liver with two regional enlarged lymph nodes. Endoscopic retrograde cholangiopancreaticography (ERCP) guided biopsy and stenting were performed. Biopsy came out as high grade small cell carcinoma. Abdomenal computed tomography (CT) showed a large 7.9 × 5.1 cm heterogeneously enhancing mass involving the gallbladder and the surrounding liver segments, namely, segments VI, V, and IVB with well-defined irregular outlines. There were associated enlarged lymph nodes: the largest one is seen at the portocaval area measuring about 4.2 × 4.7 × 7.2 cm in AP and transverse and craniocaudal diameters, respectively. A left paraaortic lymph node (PALN) was seen of size 2.9 × 4.7 × 4.7 cm. Another enlarged lymph node is seen at the aortocaval group measuring 1.8 × 1.7 cm (Figure 1). CT chest, brain, and bone scintigraphy were found negative for distant metastasis. Clinical stage was made as cT4N1 M1. Patient was started on duplet chemotherapy cisplatin 60 mg/m^2^ D1 and etoposide 100 mg/m^2^ (EP) three cycles. Postchemotherapy CT imaging showed significant response (35–45% regression) (Figure 2), and patient underwent cholecystectomy, portahepatic lymphadenectomy, and hepatic metastasectomy. Histopathology showed small cell carcinoma of gallbladder of size 5.5 cm invading subserosa, serosa, and adjacent gallbladder. Surgical margins of liver were found negative. One retrieved lymph node was found metastatic small cell carcinoma with extensive fibrosis (Figure 3). Patient was started on carboplatin and etoposide (EC) based adjuvant chemotherapy, but patient had febrile neutropenia after first cycle for which she was hospitalized. After successful recovery, she was kept on regular follow-ups. One year later, her abdomenal CT showed recurrent progressive disease with right adrenal gland invasion and progression of PALN size (Figure 4). CT-positron emission tomography (CT-PET) showed soft tissue lesion on the right adrenal region and it showed also focal fluorodeoxyglucose (FDG) uptake (SUV max 6.5). There were also two foci of FDG uptake corresponding to the left PALN (SUV 6 and 9.4). All these lesions were consistent with metastatic disease. Patient was asymptomatic and she refused any further chemotherapy at that time. Three months later she presented with one week history of right lower arm mildly painful swelling for which she was taking NSAIDs. X-ray was done which showed aggressive bony lesion involving the distal metadiaphysis and epiphysis of the right humerus and associated with the sunburst periosteal reaction and pathological fracture (Figure 5). Biopsy was consistent with metastatic small cell carcinoma of gallbladder. Palliative radiotherapy 400 cGy × five fractions (2000 cGy) was given to right lower humerus and then she was started on second line chemotherapy (paclitaxel + carboplatin) and zoledronic acid. At time of publication, she was alive at 15 months after initial diagnosis and was receiving chemotherapy.

## 3. Discussion

Small cell carcinoma (SCC) of gallbladder is a rare disease, with an incidence of approximately 0.5–3.5% of all gallbladder malignancies [9]. Median age for SCC is 67 years (25–86) and more predilection for women. Pure SCC has been documented in 41 case reports (72%) and 28% (16 cases) as SCC combined with adenocarcinoma or squamous cell carcinoma [10]. Majority of cases of SCC have been found at advanced metastatic stage (66%). Common sites of metastasis are regional lymph nodes (70%), followed by liver (64%) and lungs (10%) [10]. In our patient, rare sites (para-aortic lymphadenopathy and isolated osseous distal humerus) of metastasis were seen. Histopathological examination is only confirmatory tool and with advent of immunohistochemical techniques the incidence of reported SCC of gallbladder is expected to be higher in future [11].

Due to the scarcity and advanced stage at time of diagnosis, there are no consensus guidelines available for SCC gallbladder management. However, surgery is the best curative option for early stage tumors. For locally advanced unresectable SCC gallbladder, multi-modality approach by neoadjuvant or adjuvant chemoradiation is alternate option with better median survival rates ranging from 3.5–13 months as our patient was treated and she was alive at 15 months after initial diagnosis [12].

In conclusion, SCC of the gallbladder is very rare entity and is often seen at advanced stages. Osseous metastases of peripheral skeleton from SCC gallbladder are rarely reported. Surgery is curative option but only for early stage tumors. Incorporation of chemotherapy along with radical resection increases.
